# Tensile Creep Behavior of Quasi-Unidirectional E-Glass Fabric Reinforced Polypropylene Composite

**DOI:** 10.3390/polym10060661

**Published:** 2018-06-13

**Authors:** Zhanyu Zhai, Bingyan Jiang, Dietmar Drummer

**Affiliations:** 1 Institute of Polymer Technology, University Erlangen-Nuremberg, 91054 Erlangen, Germany; dummer@lkt.uni-erlangen.de; 2 State Key Laboratory of High Performance and Complex Manufacturing, Central South University, Lushan South Road 932, 410083 Changsha, China; jby@csu.edu.cn

**Keywords:** tensile creep, thermoplastic composites, viscoplasticity model

## Abstract

The present work addressed the creep behavior of quasi-unidirectional E-glass fabric reinforced polypropylene composites under off-axis tensile loading. A series of creep tests were performed on the composite at three different loading stress levels. The creep response of off-axis samples of quasi-unidirectional composites under a constant loading level can be clearly observed. A phenomenological viscoplasticity model was built for describing the creep behavior of the composite. To improve the accuracy of prediction, cyclic loading-unloading tests were adopted to determine the material constants in the model. The predicted results in terms of the strains after a load over a period of time were found to be satisfactory, compared with the experimental results. In addition, same failure mechanism was found in off-axis samples under quasi-static and creep loading cases.

## 1. Introduction

Fiber reinforced thermoplastic composites (FRTCs) have been widely used in many applications. As the matrix for composites, thermoplastic polymers exhibit many advantages over thermosets, like short processing time and intrinsic recyclability [1]. The durability of FRPCs is required in their service life. The long service time in combination with the viscous properties in the thermoplastic polymers emphasize the requirement of the investigation of long-term behavior of FRTCs [2]. Creep behavior is one of the time-dependent responses of material, which is a continuous deformation under a constant load [3,4]. For advanced fiber reinforced polymer composites, fibers are usually assumed as linearly elastic and do not creep. Therefore, the creep behavior observed in composites originates from the matrix [5]. Polymers generally undergo significant creep behavior even at room temperature [6]. However, it is demonstrated that fiber reinforcement can limit the creep behavior of matrix in FRTCs [7,8]. In the past years, most of research on the creep behavior of FRTCs has mainly focused on discontinuous fiber reinforced thermoplastic composites (short fibers [7,9] and long fibers [10,11]). The experimental results showed that the creep behavior of composites is affected by the factors such as the creep behavior of matrix, the geometry, the distribution of reinforcement and the fiber-matrix interfacial properties. 

So far, some investigations have carried on the creep behavior of continuous fiber reinforced thermoplastic composites (C-FRTCs) through experimental and numerical methods [2,4,12]. Liou and Teng [4] conducted a systematic investigation of the creep behavior of unidirectional (UD) carbon fiber/nylon 6 composites in the fiber direction as well as off-axis direction at three different temperatures. They found that the composite was linear viscoelastic if the off-axis angle was smaller than 30°, whereas, nonlinear viscoelastic behavior was observed when off-axis angle was increased and subjected to high stress level. The Findley power [13] was used to describe the viscoelastic creep behavior of composites. Brauner et al. [2] carried out creep tests on C-FRTCs with respect to both loading temperature and loading stress level. The extended Burgers model was used to predict the long-term viscoelastic creep behavior of composites.

On the other hand, some phenomenological viscoplasticity models were proposed in literatures [5,14,15,16,17] to predict both rate-dependent nonlinear behavior and creep behavior of polymeric composites. It should be noted that, in contrast to metals, polymeric composites do not have the plasticity mechanical behavior. However, the macro-mechanical constitutive models for polymeric composites use the terminology that is more familiar and originally developed in viscoplasticity theory [18]. The strain rate-dependent inelastic deformation including viscous and viscoelastic parts in polymeric composites are characterized by viscoplasticity. These proposed phenomenological viscoplasticity models were developed based on the one-parameter plasticity model proposed by Sun [19]. However, as illustrated by our previous work [20], the one-parameter plasticity model overestimates the plastic strain since the model fails to identify the contributors (i.e., damage and plasticity) to nonlinearity in composites. Thus, it leads to a less accurate prediction of creep strain, especially for high creep stress levels.

The objective of this work is characterizing the tensile creep behavior of quasi-unidirectional E-glass fabric reinforced polypropylene composites (quasi-UD E-glass/polypropylene composites) both experimentally and constitutively. For this purpose, tensile creep tests were firstly performed at three different loading stress levels on quasi-UD E-glass/polypropylene composites. By taking a similar approach with the viscoplasticty model proposed by Sun et al. [21], a phenomenological viscoplasticity model was developed for quasi-UD E-glass/polypropylene composites. In this model, the plastic strains of composites were determined through cyclic loading-unloading tensile tests. It was found that the predicted creep strains of this model were in good agreement with the experimental results. In addition, the failure mechanisms of composites under quasi-static loading and creep loading were also discussed.

## 2. Modeling of Creep Behavior 

The strains of composites induced by a constant stress over a long period of time are composed of time-independent initial strains and time-dependent creep strains. In terms of the incremental formulation, it can be written as [5]

(1) $$d\varepsilon_{t}=d\varepsilon_{in}+d\varepsilon_{c}$$

where $\varepsilon_{t}$ are the total strains over a long period of time t. The initial strains $\varepsilon_{in}$ are assumed to occur instantaneously as the load is applied. $\varepsilon_{c}$ are the creep strains.

For quasi-UD E-glass/polypropylene composite under a constant stress $\sigma_{0}$, the time-independent initial strain $\varepsilon_{in}$ can be determined from a coupled damage-plasticity model proposed in our previous work [20]. According to the continuum damage mechanics (CDM) developed by Ladeveze [22], the material stiffness loss in composites is characterized by the damage variable. The emergence of permanent strains is described as plasticity theory. Thus, the axial strain increments of quasi-UD E-glass/polypropylene composites under quasi-static loading are composed of the elastic and plastic parts

(2) $$d\varepsilon_{x}=d\varepsilon_{x}^{e}+d\varepsilon_{x}^{p}=\frac{d\sigma_{x}}{\left(1-D\right)E_{x}}+d\varepsilon_{x}^{p}$$

where $\varepsilon_{x}$ and $\sigma_{x}$ are axial strain and stress, respectively. $\varepsilon_{x}^{e}$ is the elastic strain, and $\varepsilon_{x}^{p}$ is the plastic strain. Subscript x refers to the loading direction. D is the damage variable. $E_{x}$ is the elastic modulus of virgin material. 

Based on the associated flow rule, the incremental plastic strains $d\varepsilon_{ij}^{p}$ can be written as

(3) $$d\varepsilon_{ij}^{p}=\frac{\partial f}{\partial \sigma_{ij}}d\lambda$$

where the stresses $\sigma_{ij}\;\left(i,j=1,2,3\right)$ refer to the principal material directions; 1-direction coincides with fiber direction; 2-direction is perpendicular to fiber direction; f is the plastic potential function proposed by Cho [23] and dλ is a proportionality factor. 

Since there is no plastic deformation in the fiber direction of quasi-UD E-glass/polypropylene composite [24], the plastic potential function in the case of plane stress state can be simplified as

(4) $$f=\sqrt{a_{2}\sigma_{22}^{2}+\sigma_{12}^{2}}+b_{2}\sigma_{22}$$

where $a_{2}$ and $b_{2}$ are the plasticity parameters.

The effective stress $\bar{\sigma }$. is defined as

(5) $$\bar{\sigma }=f$$

Following [20], it can be shown that

(6) $$d\lambda =d{\bar{\varepsilon }}_{p}$$

in which ${\bar{\varepsilon }}_{p}$ is the effective plastic strain.

For orthotropic composites subjected to off-axis tension, the effective stress and effective plastic strain are related to the applied axial stress and plastic strain as

(7) $$\bar{\sigma }=h\left(\theta \right)\sigma_{x}$$

(8) $${\bar{\varepsilon }}_{p}=\frac{\varepsilon_{x}^{p}}{h\left(\theta \right)}$$

with

(9) $$h\left(\theta \right)=\sqrt{a_{2}\sin^{4}\theta +\sin^{2}\theta \cos^{2}\theta }+b_{2}$$

**Figure 1:**
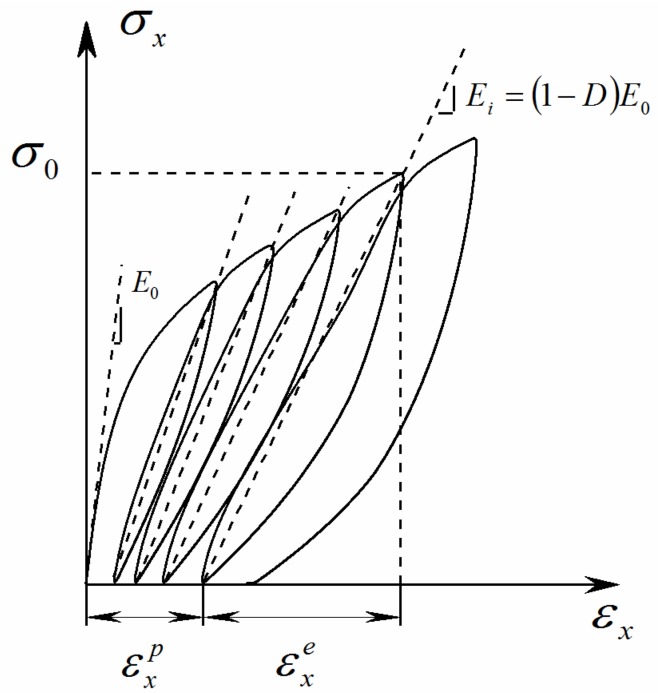
Cyclic loading-unloading tests for measuring damage variable and plastic strain [20].

Plastic strain $\varepsilon_{x}^{p}$ under the applied stress $\sigma_{x}$ can be directly obtained after the axial loading stress vanishes upon unloading, as shown in Figure 1. The master effective stress-effective plastic strain relationship can be fitted approximately with a power law [19]

(10) $${\bar{\varepsilon }}_{p}=A{\left(\bar{\sigma }\right)}^{n}$$

where A and n are parameters to be determined from experimental results. 

Finally, a coupled damage-plasticity model for quasi-UD E-glass/polypropylene composite under quasi-static loading is determined as

(11) $$\varepsilon_{x}=\frac{\sigma_{x}}{E_{0}exp\left[-{\left(\frac{\sigma_{x}h\left(\theta \right)}{\sigma_{e}}\right)}^{n_{e}}\right]}+A{\left(\sigma_{x}\right)}^{n}{\left[h\left(\theta \right)\right]}^{n+1}$$

The parameters in Equation (11) are listed in Table 2. The time-independent initial strains in Equation (1) under a constant stress $\sigma_{0}$ can be obtained as

(12) $$\varepsilon_{in}=\frac{\sigma_{0}}{E_{0}exp\left[-{\left(\frac{\sigma_{0}h\left(\theta \right)}{\sigma_{e}}\right)}^{n_{e}}\right]}+A{\left(\sigma_{0}\right)}^{n}{\left[h\left(\theta \right)\right]}^{n+1}$$

In the present paper, the creep deformation is assumed to be time-dependent plastic deformation $\varepsilon_{p}\left(t\right)$, which is composed of time-dependent viscoelastic deformation and time-dependent viscous deformation. Sun and his coworkers [21] proposed a relationship between the plastic parameter A and effective plastic strain rate ${\bar{\dot{\varepsilon }}}_{p}$ as

(13) $$A=\chi \;\;{\left({\bar{\dot{\varepsilon }}}_{p}\right)}^{m}$$

where χ and m are material constants.

Thus, the effective stress-effective plastic strain relationship for quasi-UD composite can also be rewritten as

(14) $${\bar{\varepsilon }}_{p}=\chi {\left({\bar{\dot{\varepsilon }}}_{p}\right)}^{m}{\left(\bar{\sigma }\right)}^{n}$$

and

(15) $${\bar{\dot{\varepsilon }}}_{p}=\frac{{\dot{\varepsilon }}_{p}}{h\left(\theta \right)}$$

The differential equation is represented as

(16) $${\bar{\dot{\varepsilon }}}_{p}={\left(\frac{1}{\chi }\right)}^{\frac{1}{m}}\;\;{\left(\bar{\sigma }\right)}^{\frac{-n}{m}}\;\;{\left({\bar{\varepsilon }}_{p}\right)}^{\frac{1}{m}}$$

Finally, the time-dependent strains $\varepsilon_{p}\left(t\right)$ at a constant loading stress $\sigma_{0}$ can be evaluated as

(17) $$\varepsilon_{p}\left(t\right)={\left[\frac{m-1}{m}\;\;{\left(\frac{1}{\chi }\right)}^{m}\;\;{\left[h\left(\theta \right)\right]}^{\frac{\left(1-n\right)/\left(m-1\right)}{m}}\;\;{\left(\sigma_{0}\right)}^{\frac{-n}{m}}\right]}^{\frac{m}{m-1}}\;\;t^{\frac{m}{m-1}}$$

Based on Equations (12) and (17), the sum of strains in Equation (1) can be rewritten as

(18) $$\begin{array}{c} \varepsilon_{t}=\frac{\sigma_{x}}{E_{0}exp\left[-{\left(\frac{\sigma_{x}h\left(\theta \right)}{\sigma_{e}}\right)}^{n_{e}}\right]}+9.27\times 10^{-8}{\left(\sigma_{x}\right)}^{4.5}{\left(h\left(\theta \right)\right)}^{5.5}\\ \;+{\left[\frac{m-1}{m}{\left(\frac{1}{\chi }\right)}^{m}{\left[h\left(\theta \right)\right]}^{\frac{\left(1-n\right)/\left(m-1\right)}{m}}{\left(\sigma_{0}\right)}^{\frac{-n}{m}}\right]}^{\frac{m}{m-1}}t^{\frac{m}{m-1}} \end{array}$$

The material constants in the model can be determined through cyclic loading-unloading tensile tests. Comparison with experimental results will be shown in Section 4.

## 3. Experiments

### 3.1. Materials and Samples

**Figure 2:**
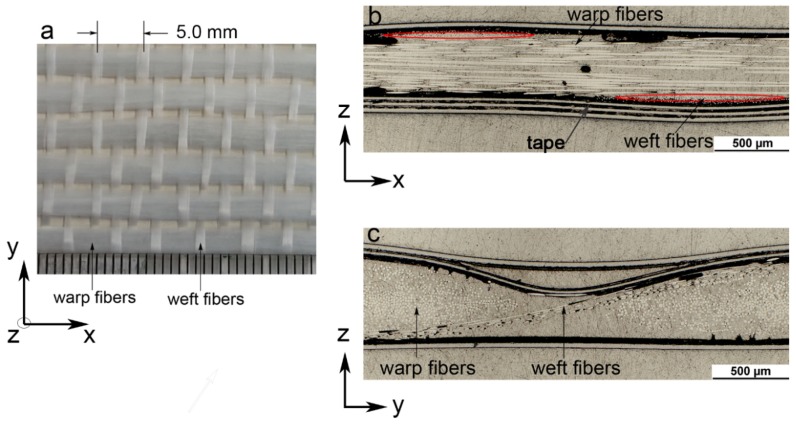
Quasi-UD E-glass fabric: (**a**) observed on the top sight (z direction), (**b**) cross-section of fabric along the warp fiber direction and (**c**) cross-section of fabric along the weft fiber direction.

The material systems investigated in the present study are quasi-UD E-glass/polypropylene composites. The reinforcement is quasi-UD E-glass fabric (as shown in Figure 2) provided by P-D Glasseiden GmbH, Germany. It consists of 92% fibers in the warp direction and 8% of fibers in the weft direction. The matrix material is copolymer polypropylene (Moplen EP500V) supplied in granule form by LyondellBasell company, which is used to produce polypropylene film. The flat film of polypropylene was extruded using a single-screw extruder (Collin E30M; screw diameter *D* = 30 mm, length-to-diameter ratio *L/D* = 25, and the width of coat-hanger die *W* = 250 mm) in combination with a chill-roll (Collin CR 136/350). The thickness and width of polypropylene film are about 0.2 mm and 210 mm, respectively.

Quasi-UD E-glass/polypropylene composites were fabricated by means of hot compression from the stacked structure, which consists of three layers of polypropylene film and two layers of quasi-UD E-glass fabric. The thickness and fiber volume fraction of composite are 0.95 ± 0.07 mm and 33.3% ± 0.5%, respectively. Correspondingly, the fiber volume fraction in the warp and weft direction are approx. 30% and 3%, respectively.

Four kinds of off-axis samples (θ =10°, 20°, 45° and 90°) were cut from quasi-UD composites using a water-cooled sawing machine. The shape and dimensions of testing samples are according to Type 2 sample of ISO 527-4. The end tabs were attached on both ends of testing samples using the Loctite 406 instant adhesive and Loctite 770 Primer. More specifically, the conventional rectangular-shaped tabs were used for 90° off-axis samples. In order to reduce the end-constrain effect induced by rigid clamps [25], the oblique end-tabs [26,27] were applied for 10°, 20° and 45° off-axis samples.

### 3.2. Experimental Procedure

Tensile creep tests were carried on 10°, 20° and 90° off-axis samples at room temperature. A testing machine of Zwick 1484 with a 100 KN load was used. The extensometer with the gauge length of 50 mm was adopted to measure strains. The testing machine was set as the position control mode when the load reached the desired creep loading stress. In this initial stage, the loading speed of 1 mm/min was used. After reaching the desired load, the testing machine was switched to the load control mode to maintain a constant loading stress. The duration of the creep test at each loading stress was 12 h if the sample did not damage before that. For each off-axis sample, the creep loading stress was set as 50%, 60% and 70% of its axial tensile strength, which were obtained from our previous work [20]. The details are listed in Table 1. In addition, to investigate damage accumulation effect on the creep strain, the elastic modulus of each testing sample after creep loading was compared with its initial elastic modulus before creep loading.

To characterize the viscoplastic behavior of quasi-UD E-glass/polypropylene composite, cyclic loading-unloading tensile tests were performed on 10°, 20°, 45° and 90° off-axis samples at the loading speed of 1 mm/min, 10 mm/min and 100 mm/min. The gauge length is 50 mm. Hence, the aforementioned crosshead speed corresponds approximately to the strain rate of 3.33 × 10^−4^, 3.33 × 10^−3^ and 3.33 × 10^−2^ 1/s, respectively. Off-axis samples were loaded and unloaded for four cycles with a gradually increasing peak stress level.

## 4. Results and Discussion

### 4.1. Creep Behavior

**Figure 3:**
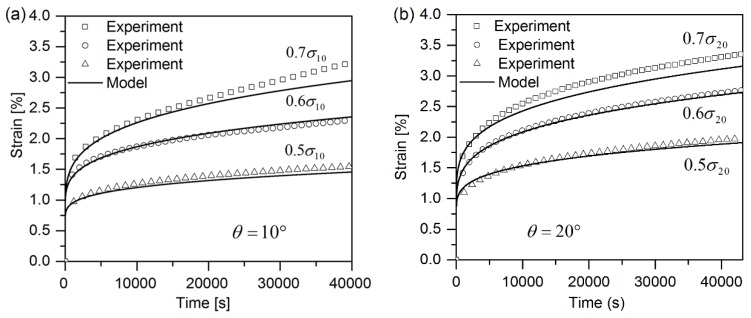

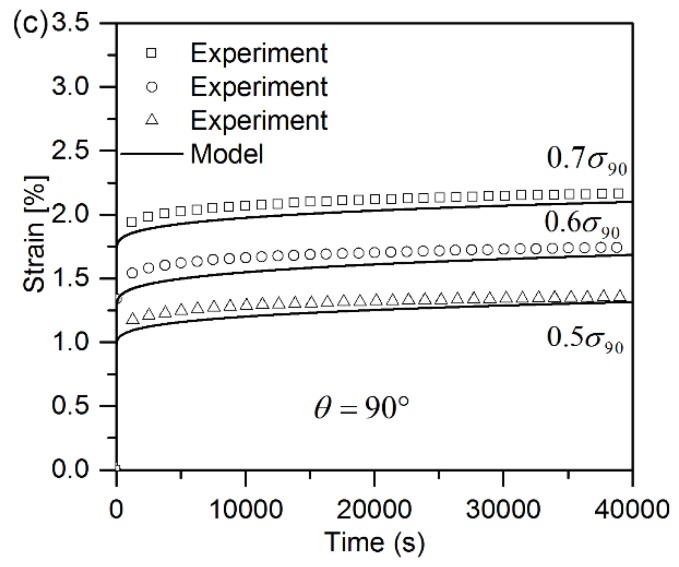
Strain-time curve for quasi-UD E-glass/polypropylene composite under off-axis loading with three creep loading levels: (**a**) 10° off-axis samples; (**b**) 20° off-axis samples and (**c**) 90° off-axis samples.

Figure 3a–c shows the experimental strain-time curves for 10°, 20° and 90° off-axis samples at three different loading levels (0.5$\sigma_{x}$, 0.6$\sigma_{x}$, 0.7$\sigma_{x}$). The creep strains for 90° off-axis samples are inappreciable compared with the initial strains, regardless of stress level. The creep curves seem to get to a steady state with an almost constant creep strain rate after a transitional period. It is as expected since the plastic deformation is small when 90° off-axis samples loaded under cyclic loading-unloading tests. Whereas, for 10° and 20° off-axis samples, the creep strains are appreciable compared to the initial strains. Moreover, the loading stress level has a significant influence on the creep strain rate of 10° and 20° off-axis samples.

**Figure 4:**
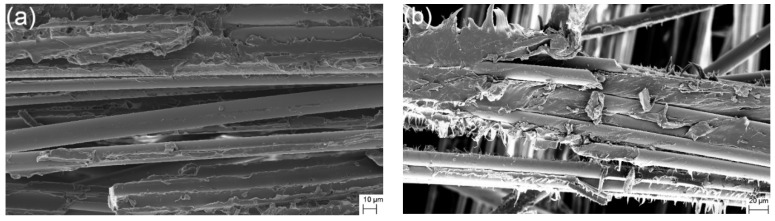
The fracture surface of 90° off-axis samples: (**a**) quasi-static loading and (**b**) creep loading.

The fracture surfaces of 90° off-axis samples under quasi-static and creep loading were comparatively observed using a scanning electron microscopy (SEM), as shown in Figure 4. For 90° off-axis samples after quasi-static tensile loading, naked fibers and fibers covered with polypropylene can be observed in Figure 4a. Meanwhile, polypropylene was split along the transverse direction directly. These failure details indicate that the fracture modes belong to adhesive and matrix failure. On the other hand, 90° off-axis samples under creep loading exhibit similar fracture behavior but more flexible/ductile behavior, as shown in Figure 4b.

### 4.2. Identification of Material Parameters for Creep Model

**Figure 5:**
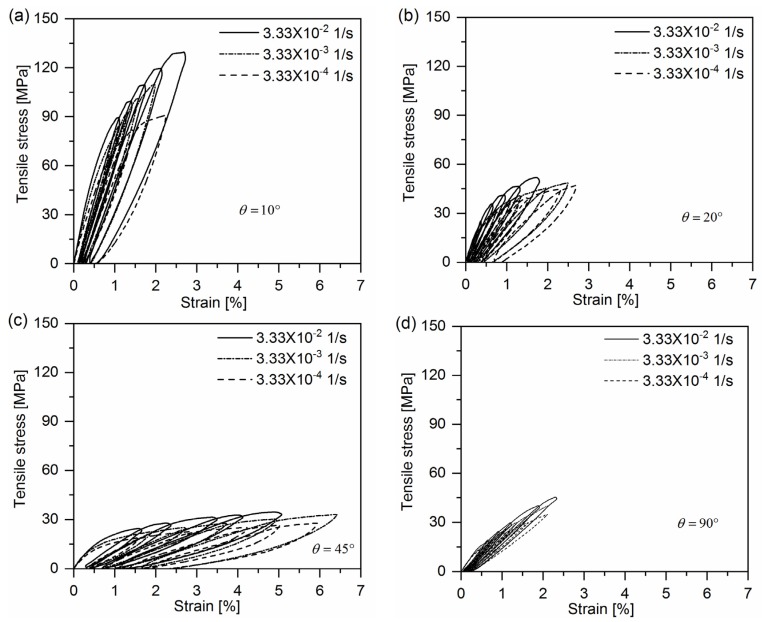
Representative cyclic loading-unloading curves for quasi-UD E-glass/polypropylene composite under different strain rates: (**a**) θ = 10°; (**b**) θ = 20°; (**c**) θ = 45° and (**d**) θ = 90°.

**Figure 6:**
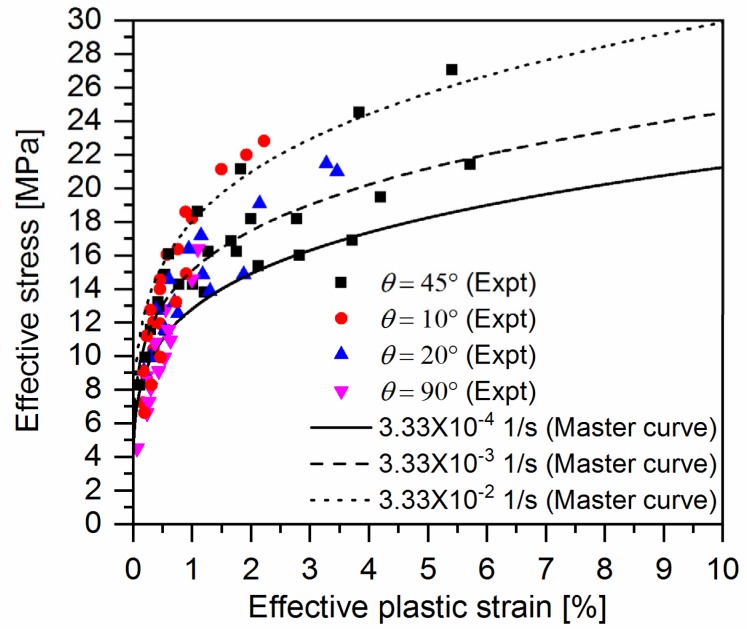
Effective stress-effective plastic curves for quasi-UD E-glass/polypropylene composite at different strain rates.

Figure 5 gives representative cyclic loading-unloading curves of quasi-UD E-glass/polypropylene composite at different strain rates. One can notice that plastic strain (permeant strain) occurs in the off-axis samples after removing the applied stress for all the strain rates. Correspondingly, the experimental data of axial loading stress-axial plastic strain of composites at different strain rates can be directly achieved from cyclic loading-unloading tests. By means of Equations (7) and (8), the coalesced effective stress-effective plastic strains at different strain rates were determined with parameters $a_{2}$ and $b_{2}$ listed in Table 2, which are shown in Figure 6. As seen in Figure 6, the master effective stress-effective plastic strain curve for quasi-UD composite at different strain rates can be fitted with Equation (13) with the parameters A and n shown in Table 2. It can be found the parameter n is independent of strain rate. 

**Figure 7:**
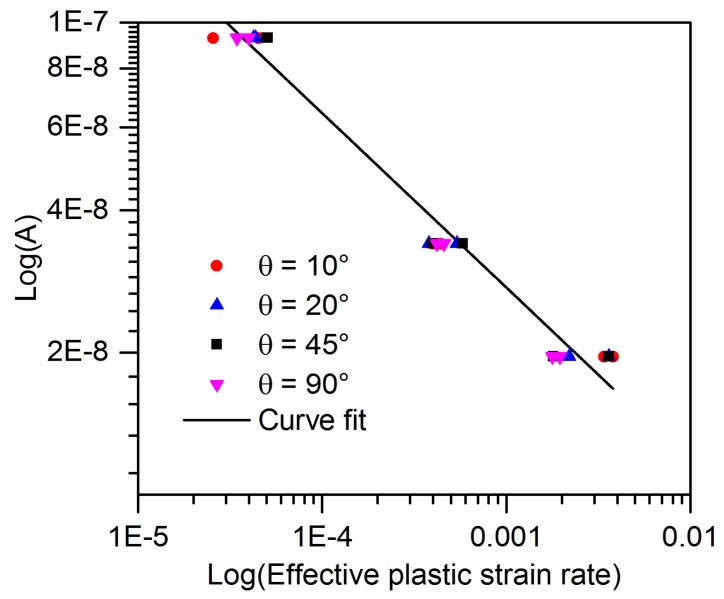
Plastic parameter *A* for quasi-UD E-glass/polypropylene composite under different effective plastic strain rate.

Figure 7 shows the plastic parameter A in Equation (10) as a function of effective plastic strain rate for the off-axis samples of quasi-UD composite on a log-log scale. Furthermore, there is appreciable scatter in the data for the effective plastic rate. The fitting parameters χ and m are listed in Table 3.

### 4.3. The Validation of Model

**Figure 8:**
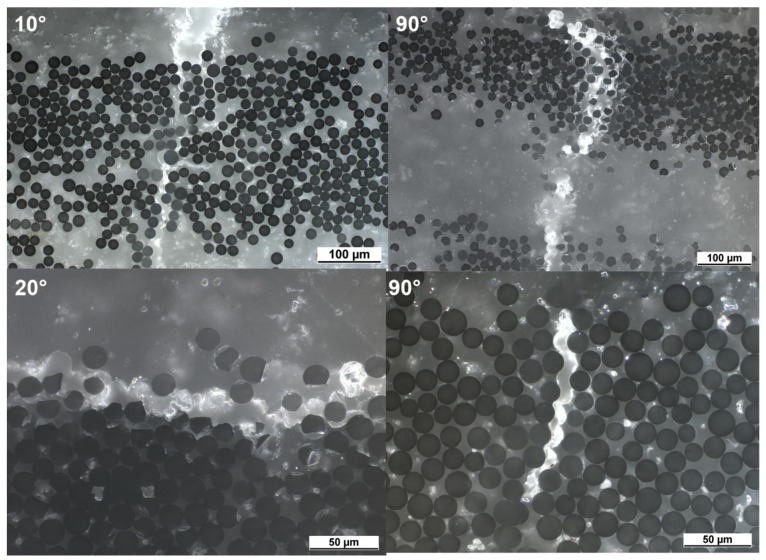
Damage events in off-axis samples after tensile creep loading.

**Figure 9:**
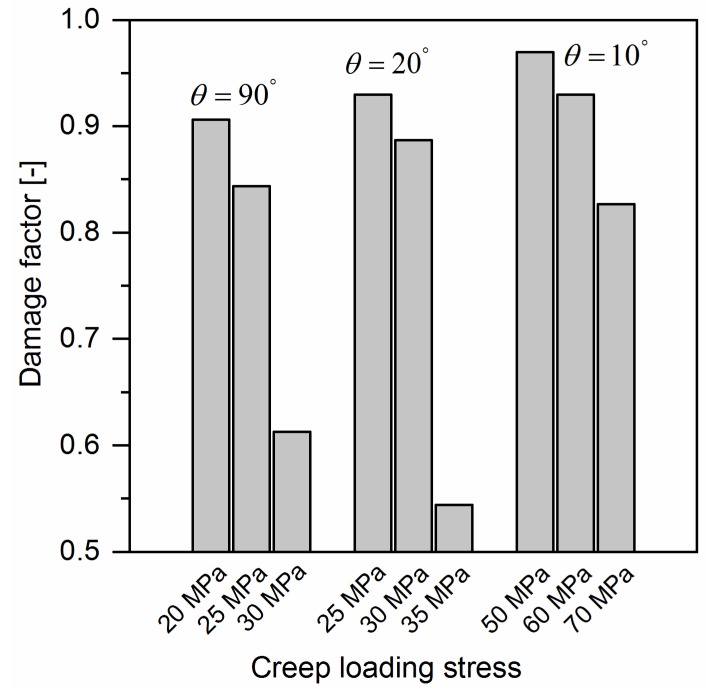
The comparison of elastic modulus of off-axis samples after creep loading with initial elastic modulus.

Based on the material parameters listed in Table 2 and Table 3 the strain-time curve of any off-axis sample of quasi-UD E-glass/polypropylene composite can be predicted by using Equation (18). The predicted results for 10°, 20° and 90° off-axis samples are plotted in Figure 3. As shown in Figure 3, a satisfactory agreement between the predicted and experimental results is observed. However, for 90° off-axis samples, Figure 3c, the experimental results are higher than the predicted results from Equation (18) at three loading levels. Same phenomenon can be found in 10° and 20° off-axis sample at the loading level of $0.7\sigma_{x}$, as shown in Figure 3a,b. It is supposed that the damage accumulations in the samples accelerated the creep strain rate, which is not taken into consideration for Equation (18). As shown in Figure 8, a large amount of damage events in the off-axis samples after tensile creep loading can be found. In addition, the damage factor ($=\frac{E_{c}}{E_{i}}$) of off-axis samples under three different creep loading levels is shown in Figure 9, which reflects the reduction in the initial elastic modulus $E_{i}$ of off-axis samples after creep loading. As seen, the elastic modulus of off-axis samples ($E_{c}$) after creep loading is lower compared to the initial elastic modulus ($E_{i}$). Thus, the deviation between the experimental and predicted results in Figure 3 may be attributed to the damage accumulations in samples during creep loading. 

## 5. Conclusions

The creep behavior of quasi-UD E-glass/polypropylene composites was investigated both experimentally and constitutively in this work. First of all, creep behavior was clearly observed for all the off-axis samples under a constant off-axis tensile loading. For 10° and 20° off-axis samples, the magnitude of creep strain became larger with increasing creep loading stress. The transient creep response was dominant in quasi-UD E-glass/polypropylene composite. The creep strain rate decreased with increasing creep strain. A phenomenological viscoplasticitiy model for quasi-UD E-glass/polypropylene composites was developed, in which the creep strain was assumed to be time-dependent plastic deformation. The material constants in the creep model were determined by using cyclic loading-unloading tensile tests. The predicted results agreed with the experimental results well. The minor deviation between the experimental and predicted creep behavior at high creep loading level may be attributed to the damage accumulations in the samples. Same failure mechanism was found in off-axis samples under quasi-static and creep loading.

## Figures and Tables

**Table 1:** Tensile creep testing plan for off-axis samples of quasi-UD E-glass/polypropylene composite.

Off-Axis Sample	Creep Loading Stress (MPa)	Loading Time (h)
10°	20, 25, 30	12
20°	25, 30, 35	
90°	50, 60, 70	

**Table 2:** Plastic parameters for quasi-UD E-glass/PP composite at three different strain rates.

Strain Rate (1/s)	Material Constants (Equation (9))		Plastic Parameters (Equation (10))	
	$a_{2}$	$b_{2}$	A ((MPa)*^−n^*)	n
3.33 × 10^−4^	0.08	0.05	9.27 × 10^−8^	4.5
3.33 × 10^−3^			3.36 × 10^−8^	
3.33 × 10^−2^			1.96 × 10^−8^	

**Table 3:** Material parameters for quasi-UD E-glass/polypropylene composite in Equation (14).

χ	−0.36
m	−8.7
